# Using SiO_2_-Supported MnO_2_@Fe_2_O_3_ Composite to Catalytically Decompose Waste Drilling Fluids Through Fenton-like Oxidation

**DOI:** 10.3390/ma17225540

**Published:** 2024-11-13

**Authors:** Tie Geng, Jiaguo Yan, Bin Li, Haiyuan Yan, Lei Guo, Qiang Sun, Zengfu Guan, Chunning Zhao, Shen Zhang, Weichao Wang

**Affiliations:** 1 Oilfield Chemicals Division, China Oilfield Services Limited (COSL), Tianjin 300450, China; gengtie@cosl.com.cn (T.G.);; 2 Tianjin Marine Petroleum Environmental and Reservoir Low-Damage Drilling Fluid Enterprise Key Laboratory, Tianjin 300450, China; 3 Shenzhen Research Institute of Nankai University, Shenzhen 518083, China; zhaochunning@mail.nankai.edu.cn (C.Z.); weichaowang@nankai.edu.cn (W.W.); 4 College of Electronic Information and Optical Engineering, Nankai University, Tianjin 300071, China

**Keywords:** waste drilling fluids, Fenton-like oxidation, SiO_2_-supported MnO_2_@Fe_2_O_3_ composite catalyst, hydrogen peroxide

## Abstract

Waste drilling fluids produced from oil extraction can cause serious harm to the ecological environment; thus, the treatment of waste drilling fluids is urgent and important to ensure the sustainability and development of the oil extraction. In this work, we used the Fenton-like reaction method to degrade waste drilling fluids with SiO_2_-supported MnO_2_@Fe_2_O_3_ composite material as a catalyst in the presence of H_2_O_2_. During the Fenton-like reaction process, the MnO_2_@Fe_2_O_3_ interface exhibits exceptional activity by facilitating the production of ·OH species with high activity and strong oxidizing properties, which degrade the organic substances in the waste drilling fluids into smaller inorganic molecules, thereby reducing its COD value. Compared to the reaction only with H_2_O_2_, after reacting with sufficient SiO_2_-supported MnO_2_@Fe_2_O_3_ catalyst for 4 h at 60 °C in the presence of H_2_O_2_, the COD value of the waste drilling fluids is reduced by 36,495 mg L^−1^, a decrease of more than 95%. This performance is significantly superior to that of the traditional Fenton reagent FeSO_4_, which reduced the COD by 32,285 mg L^−1^, a decrease of 84%. This work provides an important composite catalyst, which is practically useful for the treatment of waste drilling fluids.

## 1. Introduction

The oil and gas extraction sector is a vital energy source globally, yet its environmental footprint, particularly from waste drilling fluids, has attracted great concern. These fluids, the second most prevalent waste in oil extraction, comprise pollutants like alkanes, aromatic hydrocarbons [1,2,3], etc. Improper management can harm aquatic, terrestrial, and aerial ecosystems, impacting biodiversity, soil fertility, and human health through atmospheric contamination [1,4]. Chemical oxygen demand (COD) is a quantitative measure of the amount of oxygen required to oxidize both organic and inorganic substances in water. It is commonly used as an indicator of water quality and pollution levels, reflecting the presence of contaminants that can consume oxygen in aquatic environments. Higher COD values indicate greater levels of pollution and a higher demand for oxygen, which can negatively impact aquatic life. Consequently, reducing the COD of these wastes [5] becomes imperative.

To reduce COD values, diverse physical, chemical, and biological approaches, including thermal, bioremediation, physicochemical, and supercritical fluid treatments [6,7,8,9], have been developed during the last several decades. Physical thermal treatment is often highly energy-intensive, leading to increased operational costs and the generation of secondary gaseous pollutants that can harm the environment [10]. Although promising for large-scale applications, bioremediation processes can be slow, less efficient, and sensitive to environmental conditions, making them less reliable in various settings [11]. Physicochemical treatments often involve chemical agents that can generate additional waste and may not efficiently target all organic compounds, leading to incomplete degradation and persistently high COD values [7]. Similarly, supercritical fluid treatments require extreme conditions that are energy-intensive and complex, limiting their scalability and overall effectiveness in reducing COD in wastewater [9]. These drawbacks highlight the need for developing highly efficient strategies that specifically target and effectively decrease COD values. A more effective approach is essential for the complete decomposition of wastewater pollutants, ensuring a sustainable solution to water quality issues. Therefore, the development of a highly efficient strategy in the fluid environment to decrease COD value is crucial to essentially decompose the wastewater pollutants.

Fenton oxidation, a novel oxidation process, harnesses hydrogen peroxide (H_2_O_2_) to generate potent hydroxyl radicals (·OH), efficiently degrading organic pollutants into harmless molecules [12,13,14,15]. Its low secondary pollution, swift reaction, and effective treatment have made it a great method for organic waste treatment. Among several crucial factors, the catalyst plays a key role in influencing the efficiency of the Fenton reaction. However, traditional catalysts like FeSO_4_ generate iron sludge, causing secondary pollution [16,17,18]. Therefore, the Fenton-like strategy, with a heterogenous catalyst rather than the Fe-ion in solution, could be an alternative method to reduce the issue resulting from the Fenton reaction.

Manganese dioxide boasts a unique electronic structure featuring manganese ions on its surface that can switch between various valence states. This adaptability allows manganese dioxide to interact seamlessly with reactant molecules, fostering the formation of active intermediates with lower activation energies. Consequently, chemical reactions can proceed under more benign conditions [19]. Notably, β-MnO_2_ has demonstrated its potential to catalyze pollutants in aquatic environments [20,21].

Herein, to further enhance its catalytic performance, an interface engineering strategy was employed to modify the electronic structures of β-MnO_2_. This strategy involves creating an atomic bonding interface between *n*-type Fe_2_O_3_ and *p*-type MnO_2_, which plays a critical role in electron transfer processes essential for catalytic reactions. Doped with elements that contribute extra electrons, *n*-type Fe_2_O_3_ has a high concentration of free electrons that can move easily under an electric field. This characteristic makes it an effective material for facilitating electron transfer to adjacent semiconductors. Conversely, *p*-type MnO_2_, which has fewer valence electrons, creates holes or vacancies that act as positive charge carriers. The presence of these holes allows for the acceptance of electrons, thereby enhancing the overall conductivity and reactivity of the material. By forming a composite structure of *n*-type Fe_2_O_3_ and *p*-type MnO_2_, we enable efficient electron transfer from the Fe_2_O_3_ to the MnO_2_. This electron transfer elevates the activity of the individual catalytic sites. Additionally, SiO_2_ serves as a substrate, increasing the surface area of the active phases. Leveraging these materials’ abundant natural occurrence, low cost, and potent redox capabilities [22], the SiO_2_-supported MnO_2_@Fe_2_O_3_ composite was conceived as a cost-effective and efficient catalyst for waste drilling fluid treatment through Fenton-like reactions. We thus adopted the solid–liquid combination method to synthesize this promising composite catalyst. The synthesized compound with the MnO_2_@Fe_2_O_3_ interface exhibits exceptional activity by facilitating the production of highly reactive ·OH species, leading to a substantial reduction in the COD of waste fluids. By adjusting the catalyst amounts at room temperature, the COD decreased by over 93% in just 4 h and by more than 95% at 60 °C. This innovative work introduces a highly efficient Fenton-like catalyst composite that holds great promise for waste drilling fluid treatment.

## 2. Materials and Methods

### 2.1. Chemicals and Reagents

All solutions were prepared using ultra-pure water (18.2 MΩ cm). Manganese acetate (Mn(CH_3_OO)·4H_2_O) with a purity of 99.0% and tert-butanol A (TBA) with a purity of 99.5% were purchased from Shanghai Mirel Chemical Reagent Co., Ltd., Shanghai, China. Silica (SiO_2_) and ferric chloride (FeCl_3_·6H_2_O) were acquired from Shanghai McLin Biochemical Technology Co., Ltd., Shanghai, China. Calcium oxide (CaO) with a purity of 98.0% was obtained from Tianjin Fengchuan Chemical Reagent Technology Co., Ltd., Tianjin, China. Urea with a purity of 99.0% was sourced from Shanghai Titan Technology Co., Ltd., Shanghai, China.

### 2.2. Synthesis of Composite Catalyst

The SiO_2_-supported MnO_2_@Fe_2_O_3_ catalyst was prepared using the solid–liquid combination method. Specifically, stoichiometric ratios of Mn(CH_3_COO)_2_·4H_2_O (0.2 mol), FeCl_3_·6H_2_O (0.1 mol), SiO_2_ (0.2 mol), urea (1 mol), and ultra-pure water (1 mL) were added into the ball mill tank. The ball mill process lasts for 10 h. Then, the mixtures were calcined at 400 °C for 8 h to obtain the catalyst powder. The stoichiometry remained the same as the starting materials, and the stoichiometry molar ratio of SiO_2_, MnO_2_, and Fe_2_O_3_ is 4:4:1. A typical waste drilling fluid named Biodrill A produced at the oil extraction site was used as the target pollutant in this work.

### 2.3. Degradation Performance Measurements

A total of 60 mL of waste drilling fluids were first placed in a beaker. Calcium oxide was then added to initiate flocculation, followed by centrifugation. The supernatant was transferred to a new beaker, where a specific amount of H_2_O_2_ and catalyst were added. The mixture was stirred and allowed to react at a certain temperature for 4 h. After centrifuging again, the centrifuged solution was collected for COD measurement.

### 2.4. Characterization Methods

Powder X-ray diffraction (XRD), scanning electron microscopy (SEM), transmission electron microscopy (TEM), high-resolution TEM (HRTEM), and Brunauer–Emmett–Teller (BET) surface area analysis were employed to characterize the crystal structure and morphological properties of the catalyst. Additionally, X-ray photoelectron spectroscopy (XPS) and electron paramagnetic resonance (EPR) were used to investigate the surface valence compositions and oxygen vacancies of the material. The COD value of the solution was determined using the 5B-3C (V10) COD measuring instrument. EPR was also utilized to detect reaction intermediates during the catalytic process. Further detailed characterizations of the experiment are provided in the Characterization section of the Supporting Information.

## 3. Results and Discussion

### 3.1. Catalyst Characterization

**Figure 1:**
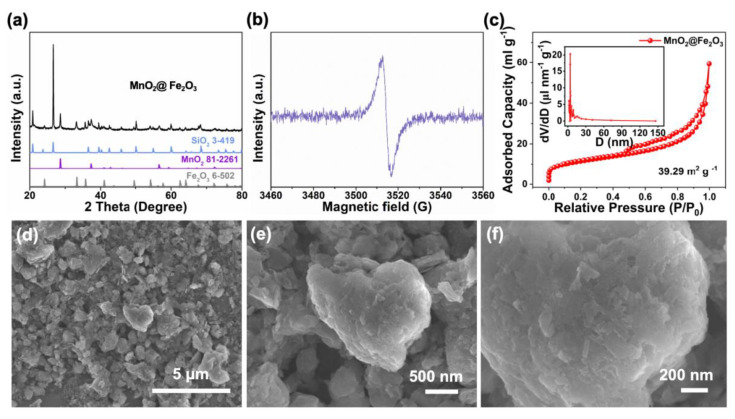
(**a**) XRD pattern, (**b**) EPR spectrum, (**c**) BET, and (**d**–**f**) SEM images with different magnifications of the magnetic field of SiO_2_-supported MnO_2_@Fe_2_O_3_ catalyst.

The crystal structure of the as-prepared catalyst was confirmed by XRD (Figure 1a); the components of the as-prepared material are SiO_2_, MnO_2,_ and Fe_2_O_3_, well corresponding to the standard cards of silicon oxide (JCPDS: 3-419), beta-pyrolusite (JCPDS: 81-2261) and hematite (JCPDS: 6-502), respectively. Additionally, the pure-phase MnO_2_ and Fe_2_O_3_ samples were also prepared, as shown in Figure S1. In addition, EPR technology was further used to characterize the oxygen feature of the material surface. Figure 1b demonstrates that a signal at 3513 G appeared, which can be assigned to the typical oxygen vacancies in the material associated with defective oxygen [23]. In contrast, the EPR results of the single component of either MnO_2_ or Fe_2_O_3_ did not show the typical signal of oxygen vacancy (Figure S2). Therefore, after the interfacing interaction between MnO_2_ and Fe_2_O_3_, oxygen vacancy may be introduced at the interface, which is beneficial for the dissociation of H_2_O_2_ to produce active oxygen free radicals. Furthermore, BET-specific surface area tests were used to measure the specific surface area, pore volume, pore size distribution of particles, and nitrogen adsorption–desorption curves of the as-prepared composite and single-phase materials. As shown in Figure 1c and Figure S3, the types of the pore size are all mesoporous. Because of the assistance of the SiO_2_ during the synthesis process, the specific surface area of the as-prepared composite material is 39.29 m^2^ g^−1^. Compared with single-phase materials, the presence of SiO_2_ only slightly increases the specific surface area of MnO_2_@Fe_2_O_3_ composite, exposing it to more catalytic active sites and thus increasing its catalytic performance. Porosity is defined as the ratio of the total pore volume within a material to its total volume. When comparing materials with the same total volume, the total pore volumes for the composite materials, MnO_2_ and Fe_2_O_3_, are 0.0757 mL g^−1^, 0.0857 mL g^−1^, and 0.0452 mL g^−1^, respectively. This indicates that MnO_2_ has the highest porosity, followed by the composite, while Fe_2_O_3_ exhibits the lowest porosity. The morphology of the catalyst was characterized by SEM. The SEM pictures under the different magnifications show that the surface morphology of SiO_2_-supported MnO_2_@Fe_2_O_3_ material is nanosphere-like with the agglomeration of nanoparticles (Figure 1d–f).

**Figure 2:**
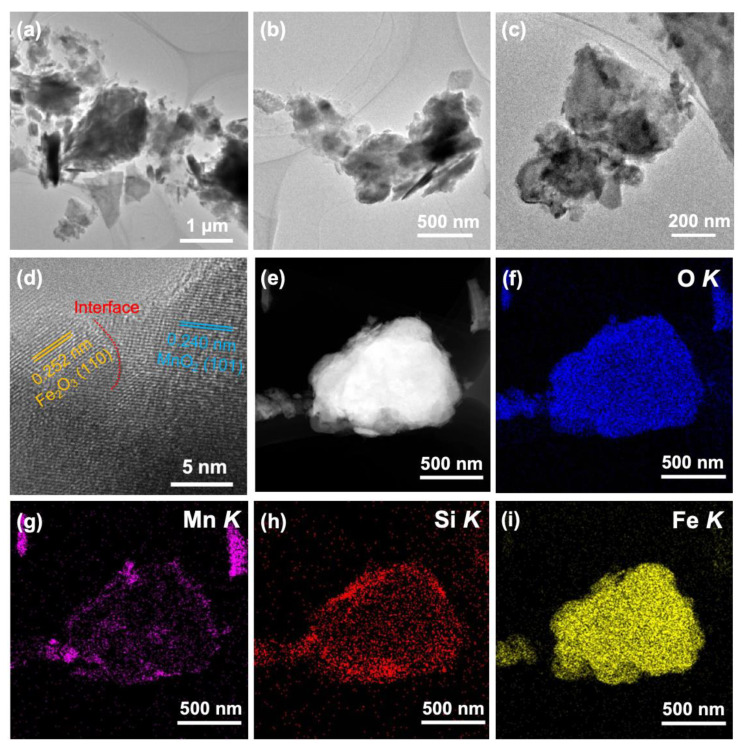
(**a**–**c**) TEM images and (**d**) HRTEM image of SiO_2_-supported MnO_2_@Fe_2_O_3_. (**e**) HAADF-STEM image and (**f**–**i**) corresponding EDS elemental mapping of SiO_2_-supported MnO_2_@Fe_2_O_3_, respectively.

The local morphology, lattice planes, and the interface information of SiO_2_-supported MnO_2_@Fe_2_O_3_ were further investigated by TEM and HRTEM. TEM images (Figure 2a–c) show that the material is uneven. The HRTEM images demonstrate that the interface of the mixed-phased occurs between the (101) of MnO_2_ and (110) of Fe_2_O_3_ (Figure 2d and Figure S4). EDS elemental mappings (Figure 2e–i) illustrate the uniform distribution of O, Mn, Si, and Fe in the catalyst, confirming its composite nature. This distribution suggests the successful formation of a composite system between Fe_2_O_3_ and MnO_2_. The morphology information of Fe_2_O_3_ and MnO_2_ was also investigated by TEM and HRTEM (Figures S5 and S6). These materials exhibit uneven morphology and demonstrate the uniform distribution of corresponding elements.

**Figure 3:**
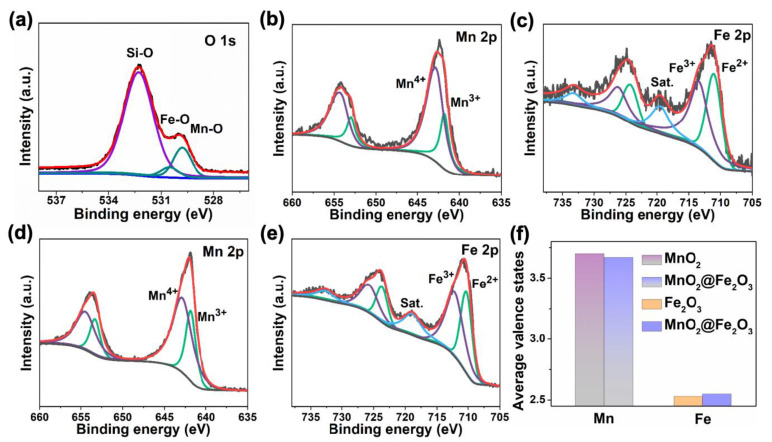
(**a**) O 1s spectra of SiO_2_-supported MnO_2_@Fe_2_O_3_, (**b**) Mn 2p spectra of MnO_2_, (**c**) Fe 2p spectra of Fe_2_O_3_, (**d**,**e**) Mn 2p and Fe 2p spectra of SiO_2_-supported MnO_2_@Fe_2_O_3_, (**f**) average valence states of Mn and Fe of the mixed- and single-phase materials.

The chemical valence states of the mixed- and single-phase of the as-prepared samples were analyzed by XPS. As shown in Figure 3a, the XPS spectrum of O 1s can be resolved into three peaks. The peaks at 529.8, 530.5, and 532.3 eV can be attributed to the oxygen species in MnO_2_, Fe_2_O_3,_ and SiO_2_, respectively [24,25].

The Fe 2p, Mn 2p, O 1s, C 1s (as peak position reference), and Si 2p were indicated in the XPS survey spectrum of the catalysts (Figure S7), consisting of the XRD and EDS mapping results. Figure 3b shows that the deconvolution of the Mn 2p_3/2_ can be decomposed into two peaks, corresponding to Mn^3+^ (641.3 eV), Mn^4+^ (642.5 eV), and a satellite peak, respectively [26,27]. The atomic ratio of Mn^3+^/Mn^4+^ in MnO_2_, according to the Mn^4+^ and Mn^3+^ peak area integrals, is 0.42. The average oxidation state (AOS) was calculated according to the equation AOS = 8.956 − 1.126∆E, in which ∆E represents the binding energy difference between the two main peaks in the Mn 3s XPS spectrum [28]. The AOS value of the catalyst is 3.70, which is lower than the theoretical value. Similarly, for the valence states of Fe in Fe_2_O_3_, the atomic ratio of Fe^2+^/Fe^3+^ is 0.89 (Figure 3c). After the two phases interface with each other, as shown in Figure 3d–f, the average valence of Mn decreases and the average valence of Fe increases compared to the single-phase systems. Hence, the charge transfer occurs at the interface of the mixed-phase system, which modifies the electronic structures at the interface.

### 3.2. Catalytic Degradation Mechanism

**Figure 4:**
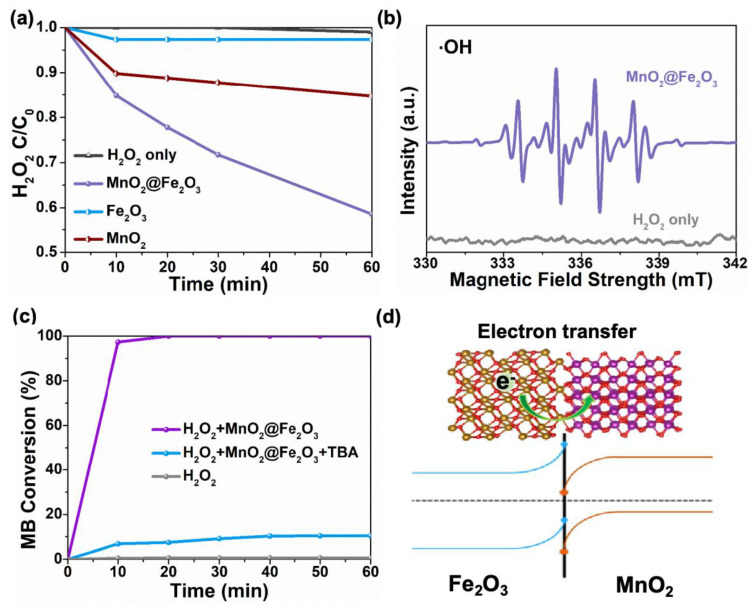
(**a**) Normalized concentration of H_2_O_2_ as a function of reaction time in the reaction of H_2_O_2_ with SiO_2_-supported MnO_2_@Fe_2_O_3_, MnO_2,_ and Fe_2_O_3_ (2 g L^−1^ catalyst, T = 25 °C with [H_2_O_2_]_0_ = 0.5 M). (**b**) EPR spectra of ·OH of catalyst/H_2_O_2_ and H_2_O_2_ system. (**c**) Degradation of MB at T = 25 °C (40 mL 0.5 M H_2_O_2_, 10 mL 55 mg L^−1^ MB in the presence of 2 g L^−1^ catalyst). (**d**) The schematic diagram of the energy band structures of the heterojunction.

First of all, the catalytic ability to degrade the waste drilling fluid pollutants of the as-prepared catalyst was tested by the experiments of H_2_O_2_ decomposition. As shown in Figure 4a, when there is no catalyst present in the system, the decomposition capacity of H_2_O_2_ is negligible. After introducing the prepared catalyst, a significant catalytic effect on H_2_O_2_ is observed. Approximately 40% of H_2_O_2_ degradation capacity is achieved at 25 °C in one hour. In contrast, the single-phase materials, including MnO_2_ and Fe_2_O_3_, exhibit much lower activity than the mixed-phase catalyst. Based on the relationship between the reaction rate constant and specific surface area, the normalized catalytic activity for the SiO_2_-supported MnO_2_@Fe_2_O_3_ and the single-phase catalysts was obtained to highlight the optimal activity of SiO_2_-supported MnO_2_@Fe_2_O_3_ (Table S1). For SiO_2_-supported MnO_2_@Fe_2_O_3_, the obtained reaction rate constant per square meter of surface area is 0.0085 min^−1^ m^−2^, significantly higher than those of MnO_2_ (0.0033 min^−1^ m^−2^) and Fe_2_O_3_ (0.0007 min^−1^ m^−2^). These results show that SiO_2_-supported MnO_2_@Fe_2_O_3_ has the best catalytic activity, indicating that even with MnO_2_ and Fe_2_O_3_ with SiO_2_ assistance, the catalytic performance of single-phase catalysts is not as good as that of composite catalysts. Therefore, the single-phase catalysts with SiO_2_ assistance were not prepared in this manuscript. The enhanced activity of SiO_2_-supported MnO_2_@Fe_2_O_3_ is mainly attributed to the effect of interfacial recombination. To further understand the enhanced catalytic activity under the interfacing engineering, the EPR spectra were used to identify the intermediates that can be generated in the waste drilling fluids/H_2_O_2_ system. Figure 4b and Figure S8 demonstrate that when only H_2_O_2_ was used in the system as the background, ·OH was not observed. The pronounced ·OH signal was detected in the presence of catalyst and H_2_O_2_. Especially compared with the single-phase materials, the interface in the mixed-phase catalyst facilitates the production of the active hydroxyl radicals.

In order to identify the active species in this catalytic degradation reaction, we selected methylene blue (MB) as a model pollutant. The degradation performance of a SiO_2_-supported MnO_2_@Fe_2_O_3_ catalyst on methylene blue is shown in Figure 4c. SiO_2_-supported MnO_2_@Fe_2_O_3_ catalyst can achieve a 97% degradation rate of methylene blue within 10 min. When tert-butanol A (TBA) was added to the reaction system as an excessive ·OH quencher, the performance of SiO_2_-supported MnO_2_@Fe_2_O_3_ catalytic degradation of methylene blue decreased significantly, which indicated that the reaction intermediate ·OH was used as a key active species for oxidative degradation of organic pollutants. During the catalytic degradation process, ·OH, as a highly active and strong oxidizing free radical, can degrade organic substances in the waste drilling fluids into small-molecule inorganic substances, thereby reducing its COD value.

To further understand the inner physics of the promotion effect by the interfacing engineering, we analyzed the energy band structures of MnO_2_ and Fe_2_O_3_. The enhanced catalytic activity of SiO_2_-supported MnO_2_@Fe_2_O_3_ is primarily attributed to the effects of interfacial electron transfer. The work functions of MnO_2_ and Fe_2_O_3_ are ~6.67 eV and ~5.9 eV, respectively [29,30]. This difference establishes a potential energy gradient at the interface when the two materials come into contact, driving electron transfer from the *n*-type Fe_2_O_3_ to the *p*-type MnO_2_ (Figure 4d). Upon contact, the Fermi levels of the two materials equilibrate, resulting in a flattening of the energy bands. This electron transfer causes the electrons to accumulate at the interface, leading to the formation of an energy barrier that stabilizes the heterojunction. The accumulation of electrons at the interface modifies the valence states of Mn in the composite. This shift results in lower valence states compared to the single-phase MnO_2_, altering its electronic properties and enhancing its catalytic performance. The formation of the heterojunction influences the interaction strength between the catalyst surface and reaction intermediates. The presence of accumulated electrons affects the surface charge distribution, facilitating more effective bonding with organic pollutants and improving the overall reactivity. The heterojunction acts as an active interface that promotes the generation of reactive radicals, which is crucial for degrading organic pollutants. The electron-rich environment at the interface enhances the ability of the catalyst to participate in redox reactions. By adjusting the electronic structure of MnO_2_, Fe_2_O_3_ helps optimize the coordination environment at the interface. This optimization ensures that active radicals are generated efficiently and that their interaction with organic pollutants is maximized, improving the degradation efficiency.

### 3.3. Catalytic Degradation of Waste Drilling Fluids

We used Fenton-like oxidation technology to catalyze H_2_O_2_ to degrade waste drilling fluids and conducted a series of experiments by optimizing experimental conditions, including reaction temperature, H_2_O_2_ concentration, and catalyst dosage.

**Figure 5:**
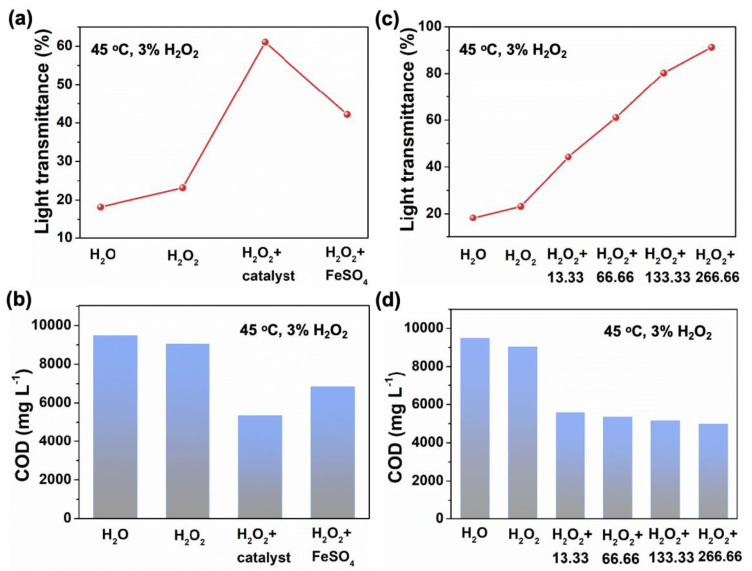
Light transmittance results of catalyst-catalyzed degradation of waste drilling fluids at T = 45 °C (**a**) with different catalysts and (**c**) with different amounts of SiO_2_-supported MnO_2_@Fe_2_O_3_ catalyst. COD results of catalytic degradation of waste drilling fluids (**b**) with different catalysts and (**d**) with different amounts of SiO_2_-supported MnO_2_@Fe_2_O_3_ catalyst. The numbers in the x-axle in (**c**,**d**) represent the catalyst amounts during this reaction.

At 45 °C and the mass fraction of H_2_O_2_ in the waste drilling fluids was 3%, we conducted degradation experiments on the waste drilling fluids and reflected its degradation performance through the values of light transmittance and COD. As shown in Figure 5a and Figure S9, when the calculated amount of water is added to the waste drilling fluids as a blank control, the light transmittance of the waste drilling fluids is 18.2. After adding an equal amount of H_2_O_2_ to the waste drilling fluids, the light transmittance increases to 23.2. When H_2_O_2_ and SiO_2_-supported MnO_2_@Fe_2_O_3_ catalysts were added to the waste drilling fluids at the same time, the light transmittance further increased to 61.1. The increase in light transmittance indicates that the colored substances in the waste drilling fluids have been effectively degraded. The degradation performance of waste drilling fluids is further reflected by the reduction in COD value. The greater the COD value decreases, the more organic matter in the waste drilling fluids has been degraded. When only the calculated amount of water is added to the waste drilling fluids, its COD value is 9480 mg L^−1^ (Figure 5b). After adding an equal amount of H_2_O_2_ to the waste drilling fluids, its COD value dropped to 9030 mg L^−1^. When there are both H_2_O_2_ and SiO_2_-supported MnO_2_@Fe_2_O_3_ catalysts in the waste drilling fluids, the COD value is reduced to 5340 mg L^−1^, which is significantly better than the same amount of traditional Fenton reagent FeSO_4_ (6830 mg L^−1^), demonstrating that the SiO_2_-supported MnO_2_@Fe_2_O_3_ catalyst has a better degradation effect on waste drilling fluids than Fenton’s reagent.

We further adjusted the amount of catalyst, and the light transmittance of the waste drilling fluids by only adding the water control group was 18.2 (Figure 5c). After adding an equal amount of H_2_O_2_, compared with the system without adding catalyst, in the systems adding 13.33 mg, 66.66 mg, 133.33 mg, and 266.66 mg catalyst, respectively, the light transmittance after degrading the waste drilling fluids increased from the initial 23.2 to 44.2, 61.1, 80.2, and 91.2, indicating that the degradation effect of colored substances in waste drilling fluids was better with the increase in catalyst dosage. As the amount of catalyst increases, the COD value continues to decrease, which is consistent with the increase in light transmittance (Figure 5d). The COD value after degrading the waste drilling fluids dropped from the initial 9480 mg L^−1^ to 4967 mg L^−1^, and the COD removal rate of organic matter in the waste drilling fluids was 47.6%.

In addition to the light transmittance and COD, we pay attention to the viscosity parameters of the waste drilling fluid pollutants after and before catalysis. As summarized in Table 1, the apparent viscosity (AV), plastic viscosity (PV), and shear force (YP) were tested to comprehensively evaluate the breaking ability of the catalytic process. Evidently, the viscosity of the waste drilling fluid is reduced largely by introducing the catalyst with the H_2_O_2._ The YP reached a drop of two orders of magnitude. This good ability to lower the viscosity of the waste drilling fluids decreases the pressure of the discharge, storage, and transportation in practical use.

**Figure 6:**
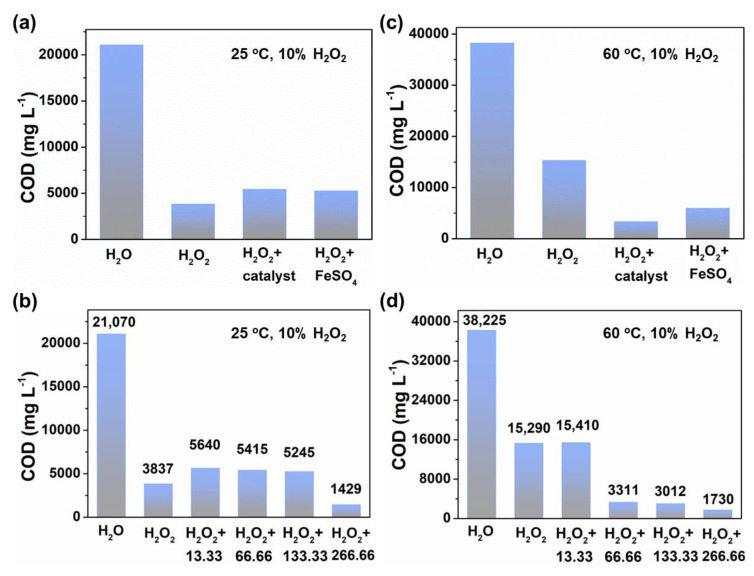
COD results of catalyst-catalyzed degradation of waste drilling fluids at (**a**) 25 °C and (**c**) 60 °C, respectively. COD results of catalytic degradation of waste drilling fluids using different amounts of catalysts at (**b**) 25 °C and (**d**) 60 °C, respectively. The numbers in the x-axle in (**b**,**d**) represent the catalyst amounts during this reaction.

In addition, we controlled the concentration of H_2_O_2_ in the reaction system to remain the same and changed the reaction temperature. At room temperature of 25 °C, add the calculated amount of H_2_O_2_ to the waste drilling fluids to make the H_2_O_2_ mass fraction 10%. Add the same amount of water to the waste drilling fluids as a control experiment, and evaluate the degradation performance of the catalyst on the waste drilling fluids through the COD value. Figure 6a shows that when H_2_O_2_ and catalyst are added to the system, the COD value of the waste drilling fluids decreases significantly. We further adjusted the amount of SiO_2_-supported MnO_2_@Fe_2_O_3_ catalyst. Figure 6b exhibits that compared with no catalyst, the COD value of waste drilling fluids with sufficient catalyst added at room temperature 25 °C decreased by 19,640.5 mg L^−1^, and the COD removal rate exceeded 93%.

When the concentration of H_2_O_2_ in the reaction system is kept constant and the reaction temperature is changed to 60 °C, the degradation effect on the waste drilling fluids is shown in Figure 6c. There is a significant difference in the COD value of waste drilling fluids without a catalyst at 60 °C and room temperature of 25 °C. The reason is that an increase in temperature may cause more water to evaporate, increase the solution concentration, and thereby increase the COD value of the waste drilling fluids. When H_2_O_2_ exists in the system, the COD value of the waste drilling fluids is lower than the initial value. When H_2_O_2_ and catalyst exist at the same time, the COD value of the waste drilling fluids further decreases. Moreover, the catalytic degradation performance of SiO_2_-supported MnO_2_@Fe_2_O_3_ catalyst is significantly superior to the same amount of traditional Fenton reagent FeSO_4_. As the amount of catalyst increases, its catalytic degradation performance on waste drilling fluids becomes better (Figure 6d). Sometimes, the COD is higher for catalysts in comparison to no catalysts, which may be due to the fact that the catalyst is not completely filtered out after the catalytic reaction is completed, so the solution inevitably contains a small amount of catalyst. Residual catalysts in the solution can potentially influence the COD value. These catalysts can adsorb organic pollutants during the reaction and later release them back into the solution. It can lead to an increase in COD, counteracting the intended degradation effects. In the waste drilling fluids system with a sufficient amount of catalyst, the COD value dropped by 36,494.5 mg L^−1^ after the reaction was completed, and the COD removal rate exceeded 95%, which can be comparable to other reported work (Table S2).

Together, either MnO_2_ or Fe_2_O_3_ alone fails to efficiently decompose the oil waste drilling solution. Importantly, the interface between these two phases was found to play a key role in boosting the degradation of the pollutant and thus decreasing COD value. The improvement could be ascribed to the interface charge transfer from Fe_2_O_3_ to MnO_2_. In further work, more interface refinements, including different surface alignments, morphology, interfacial charge states, etc., should be carried out to further increase the catalytic performance in the Fenton-like reaction.

## 4. Conclusions

In summary, the SiO_2_-supported MnO_2_@Fe_2_O_3_ catalyst has been effectively developed for the treatment of waste drilling fluids using Fenton-like reaction technology. The interface formed between MnO_2_ and Fe_2_O_3_ demonstrates remarkable catalytic activity due to enhanced electron transfer facilitated by the formation of a heterojunction. This catalyst generates highly active and potent oxidizing species during the catalytic decomposition of hydrogen peroxide, leading to the efficient degradation of organic pollutants present in the waste drilling fluids. Experimental results show a significant reduction in COD values. After 4 h of reaction at room temperature (25 °C) with the SiO_2_-supported MnO_2_@Fe_2_O_3_ catalyst, the COD value decreased by 19,641 mg L^−1^, achieving a reduction of over 93% compared to the untreated waste drilling fluids. Furthermore, when the reaction was conducted at an elevated temperature of 60 °C, the COD value decreased by 36,495 mg L^−1^, exceeding 95% reduction and outperforming the same quantity of commercial Fenton reagent FeSO_4_. This work highlights the potential of the SiO_2_-supported MnO_2_@Fe_2_O_3_ catalyst as an effective and sustainable solution for the large-scale processing of waste drilling fluids, offering significant improvements in organic pollutant degradation.

## Figures and Tables

**Table 1:** The viscosity parameters (AV, (AV = 1/2 × ∮600), PV, (PV = ∮600 − ∮300), and YP, (YP = 0.4788 × (∮300 − PV)), light transmittance, and COD values of the degradation of waste drilling fluids catalyzed by different catalysts. Samples 1–6# represent the blank reference, with H_2_O_2_, with H_2_O_2_ and 13.33 mg catalyst, with H_2_O_2_ and 66.66 mg catalyst, with H_2_O_2_ and 133.33 mg catalyst, with H_2_O_2_ and 266.66 mg catalyst, respectively.

Sample	∮600	∮300	AV (mPa·s)	PV (mPa·s)	YP (Pa)	Light Transmittance (%)	COD (mg L^−1^)
1#	11.49	17.51	8.73	6.03	2.63	18.17	9480
2#	3.01	5.03	2.52	2.01	0.48	23.16	9030
3#	2.42	4.41	2.22	2.03	0.19	44.23	5567
4#	2.23	4.33	2.14	2.12	0.10	61.04	5340
5#	2.04	3.90	1.93	1.91	0.05	80.22	5145
6#	1.93	3.72	1.82	1.84	0.05	91.23	4967

## Data Availability

The data presented in this study are available on request from the corresponding author.

## Supplementary Materials

The following supporting information can be downloaded at: https://www.mdpi.com/article/10.3390/ma17225540/s1, Text S1: Characterization Scanning electron microscopy (SEM) was performed on a Hitachi Model SU8010 system. Text S2: Free Radical Detection In a typical EPR experiment, 0.01 g catalyst was added to a 10 mL H_2_O_2_ (0.5 M) solution. Figure S1: XRD pattern of (a) MnO_2_ and (b) Fe_2_O_3_, respectively. Figure S2: EPR spectrum of (a) MnO_2_ and (b) Fe_2_O_3_, respectively. Figure S3: N2 adsorption-desorption isotherms of (a) MnO_2_ and (b) Fe_2_O_3_, respectively. Figure S4: HRTEM image of MnO_2_@Fe_2_O_3_. Figure S5: (a) SEM image and (b) TEM image of Fe_2_O_3_. (c) HAADF-STEM image and (d–f) corresponding EDS elemental mapping of Fe_2_O_3_, respectively. The table displays content of Fe and O in Fe_2_O_3_. Figure S6: (a) SEM image and (b) TEM image of Fe_2_O_3_. (c) HAADF-STEM image and (d–f) corresponding EDS elemental mapping of MnO_2_, respectively. The table displays content of Mn and O in MnO_2._ Figure S7: XPS survey spectrum of (a) MnO_2_, (b) Fe_2_O_3_ and (c) MnO_2_@Fe_2_O_3_, respectively. Figure S8: EPR spectra of OH of catalyst/H_2_O_2_ when the reaction was carried out for 15 s, respectively. Figure S9: At 45 °C, when the mass fraction of H_2_O_2_ in the drilling fluid is 3%, the picture after the degradation reaction of the drilling fluid. Table S1: The normalized catalytic activity (reaction rate constant per square meter of surface area) for the SiO_2_-supported MnO_2_@Fe_2_O_3_, MnO_2_ and Fe_2_O_3._ Table S2: Comparison of the optimal COD removal rate for drilling fluids between this work and other reported work [31,32,33].
