# Supplementary material for: Using SiO2-Supported MnO2@Fe2O3 Composite to Catalytically Decompose Waste Drilling Fluids Through Fenton-like Oxidation
Source: Materials (Basel). 2024 Nov 13;17(22):5540. doi: 10.3390/ma17225540 (PMC11595276; doi:10.3390/ma17225540)
Supplement: Supplementary file 1 [file materials-17-05540-s001.zip › materials-3278893-supplementary.pdf]

Supporting Information

for

Using SiO2-Supported MnO2@Fe2O3 Composite to Catalytically Decompose Waste Drilling Fluids Through Fenton-like Oxidation

Tie Geng [1,2,†], Jiaguo Yan [1,2,*,†], Bin Li [1,2], Haiyuan Yan [1,2], Lei Guo [1,2], Qiang Sun [1,2], Zengfu Guan [1,2], Chunning Zhao [3,4], Shen Zhang [3,4,*,†] and Weichao Wang [3,4]

[1] *Oilfield Chemicals Division, China Oilfield Services Limited (COSL), Tianjin 300450, China*

[2] *Tianjin Marine Petroleum Environmental and Reservoir Low-Damage Drilling Fluid Enterprise Key Laboratory, Tianjin 300450, China*

[3] *Shenzhen Research Institute of Nankai University, Shenzhen 518083, China*

[4] *College of Electronic Information and Optical Engineering, Nankai University, Tianjin 300071, China*

* *Correspondence: yanjg5@cosl.com.cn (J.Y.); 1120210136@mail.nankai.edu.cn (S.Z.)*

† These authors contributed equally to this work.

Section S1. Supporting Materials and Methods

Text S1. Characterization

Scanning electron microscopy (SEM) was performed on a Hitachi Model SU8010 system. Transmission electron microscopy (TEM), high angle annular dark field scanning TEM (HAADF-STEM) and energy dispersive X-ray spectroscopy (EDS) were carried out on an FEI Tecnai G2 F30 transmission electron microscope. Powder X-ray diffraction (XRD) patterns were recorded on a desktop X-ray diffractometer (RIGAKU-Miniflex 600) with Cu Kα radiation. X-ray photoelectron spectroscopy (XPS) measurements were performed on a Thermo ESCALAB 250 spectrometer using nonmonochromatic Al Kα X-ray as the excitation source and choosing C 1s (284.8 eV) as the reference line. Hydroxyl radical (·OH) and oxygen vacancies were detected by electron paramagnetic resonance (EPR, CIQTEK EPR200-Plus). Nitrogen adsorption and desorption isotherms were measured at 77 K using a Micromeritics ASAP 2020 system. The samples were degassed at 150 °C for 10 h before the measurements. Specific surface areas were calculated from the adsorption data using Brunauer-Emmett-Teller (BET) equation. The chemical oxygen demand (COD) of the drilling fluid was measured using a 5B-3C(V10) COD ammonia nitrogen dual parameter measuring instrument.

Text S2. Free Radical Detection

In a typical EPR experiment, 0.01 g catalyst was added to a 10 mL H_2O_2 (0.5 M) solution. Then, this solution was filtered through a Naflon membrane of 0.22 μm and 1

ml filtrate was immediately injected into 1 ml DMPO (Sigma Aldrich, 100 ppm). The obtained solution was transferred to a 100 μL capillary tube, which was then fixed in the resonant cavity of the spectrometer. H_2O_2 (0.5 M) solution was also measured using the same procedure as the reference.

Section S2. Figures and Tables

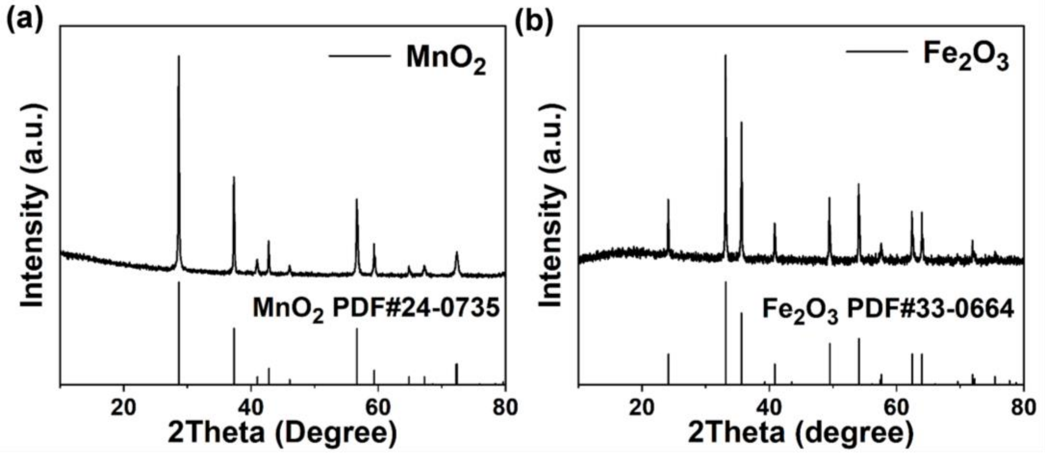

Figure S1. XRD pattern of (a) MnO_2 and (b) Fe_2O_3, respectively.

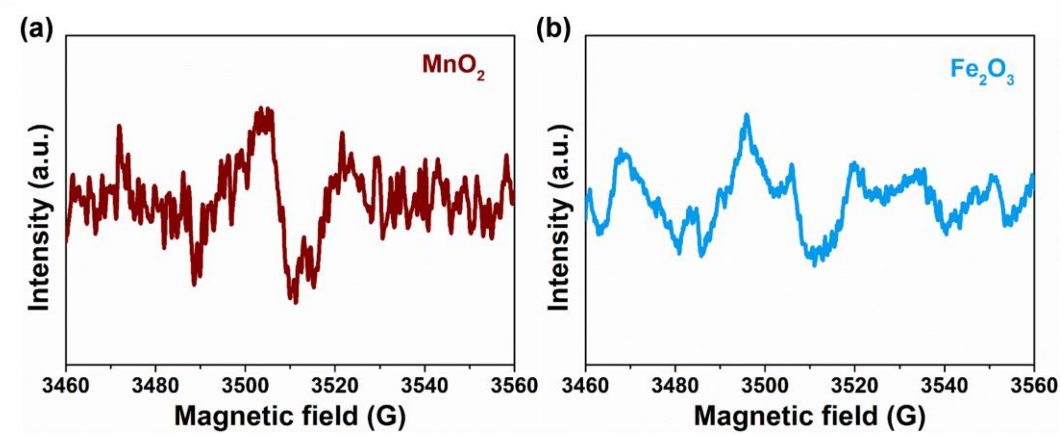

Figure S2. EPR spectrum of (a) MnO_2 and (b) Fe_2O_3, respectively.

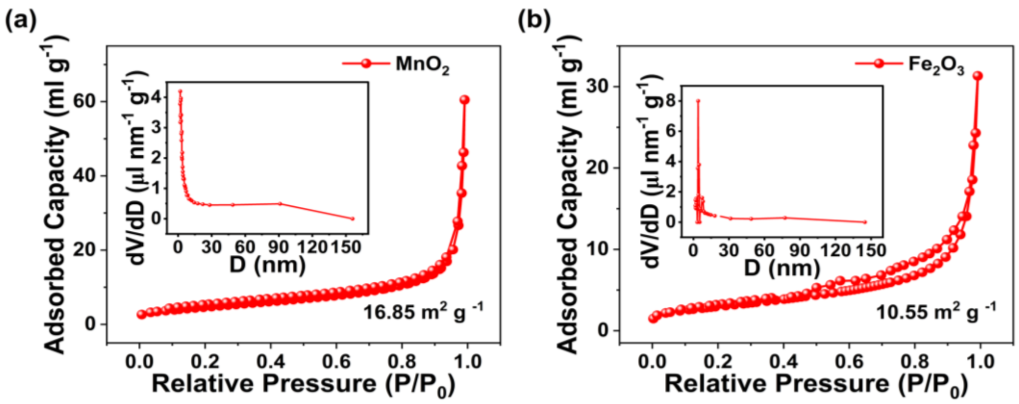

Figure S3. N_2 adsorption-desorption isotherms of (a) MnO_2 and (b) Fe_2O_3, respectively.

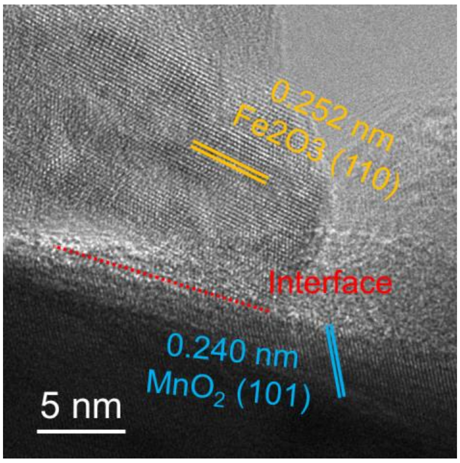

Figure S4. HRTEM image of $MnO_2@Fe_2O_3$.

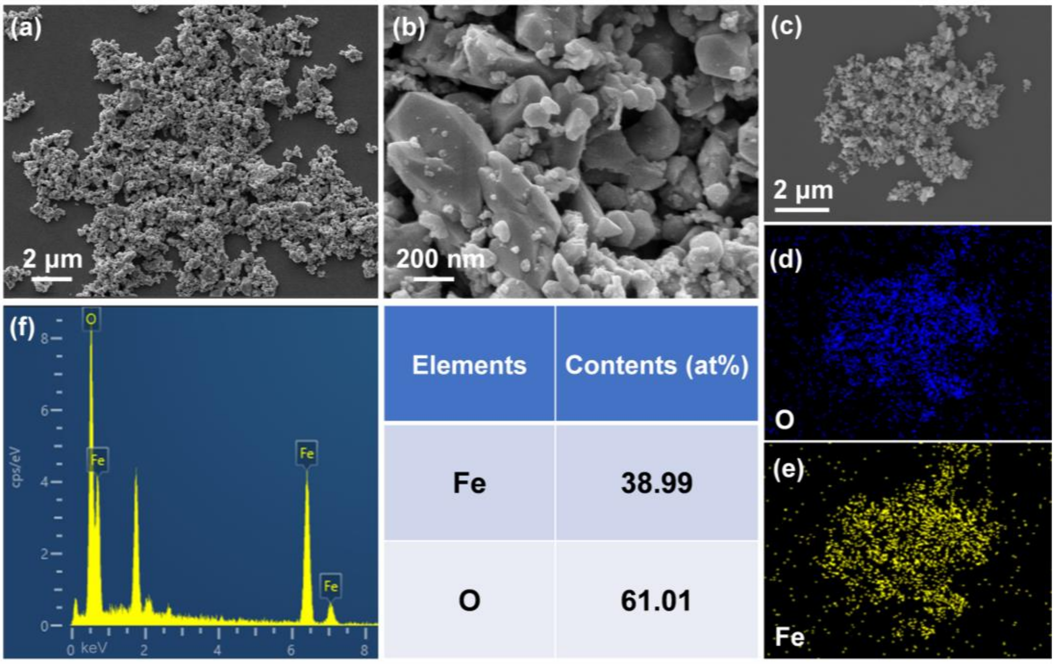

Figure S5. (a) SEM image and (b) TEM image of Fe_2O_3. (c) HAADF-STEM image and (d–f) corresponding EDS elemental mapping of Fe_2O_3, respectively. The table displays content of Fe and O in Fe_2O_3.

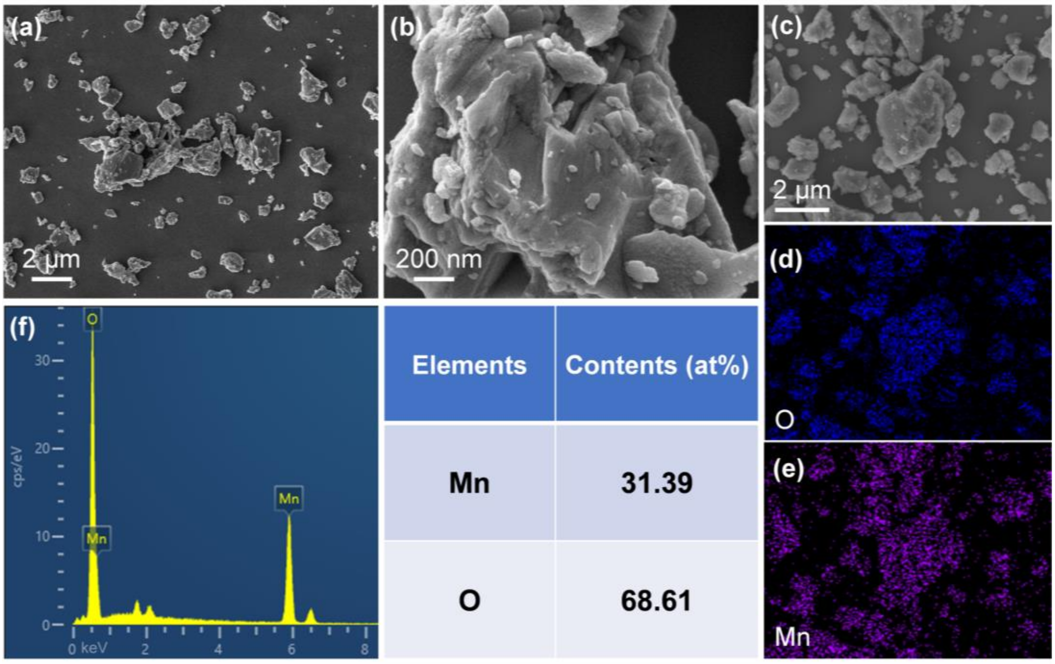

Figure S6. (a) SEM image and (b) TEM image of Fe_2O_3. (c) HAADF-STEM image and (d–f) corresponding EDS elemental mapping of MnO_2, respectively. The table displays content of Mn and O in MnO_2.

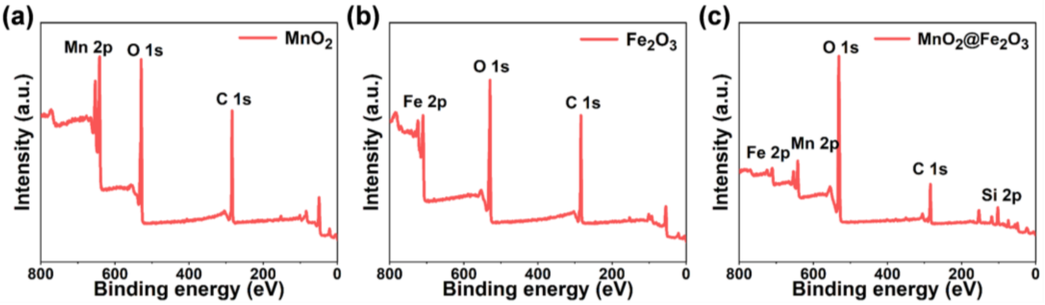

Figure S7. XPS survey spectrum of (a) MnO_2, (b) Fe_2O_3 and (c) $MnO_2@Fe_2O_3$, respectively.

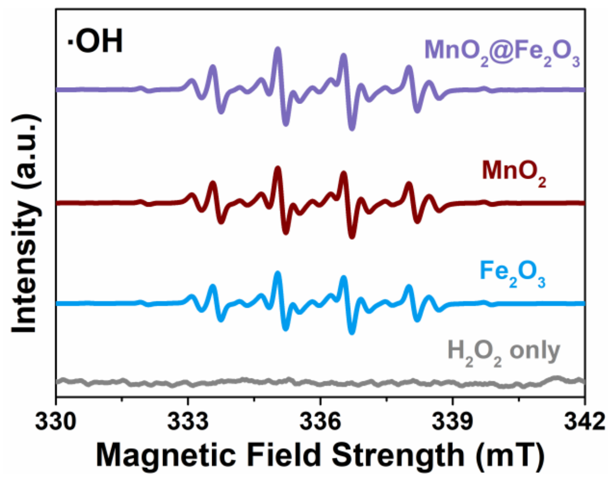

Figure S8. EPR spectra of ·OH of catalyst/H_2O_2 when the reaction was carried out for 15 s, respectively.

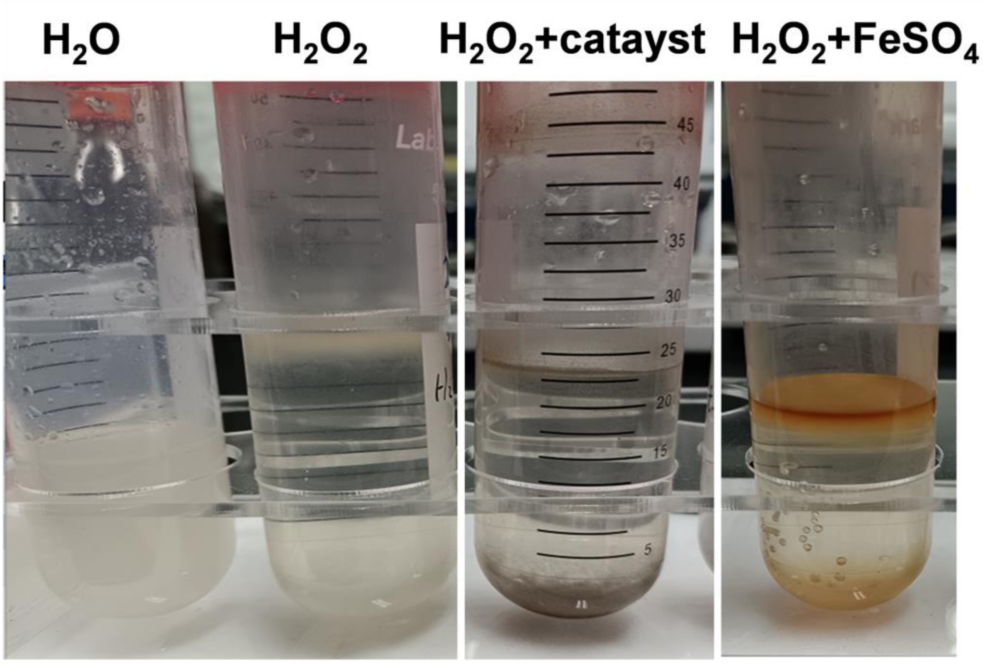

Figure S9. At 45 °C, when the mass fraction of H_2O_2 in the drilling fluid is 3%, the picture after the degradation reaction of the drilling fluid.

Table S1. The normalized catalytic activity (reaction rate constant per square meter of surface area) for the SiO_2-supported MnO_2@Fe_2O_3, MnO_2 and Fe_2O_3.

Samples	Normalized reaction rate constant
SiO_2-supported MnO_2@Fe_2O_3	0.0085 min^{-1} m^{-2}
MnO_2	0.0033 min^{-1} m^{-2}
Fe_2O_3	0.0007 min^{-1} m^{-2}

Table S2. Comparison of the optimal COD removal rate for drilling fluids between this work and other reported work.

Catalysts	COD removal rate (%)	Ref.
**$MnO_2@Fe_2O_3$**	**95**	**This work**
polyacrylamide, polyaluminum chloride and $FeSO_4$	72	[31]
adsorbent O-HTACC-g-CD	96	[32]
SQ6-1 strain	40.26	[33]

References

1. Zhang, J.; Liu, X.; Li, Y.; Chang, X.; Zhang, J.; Chen, G. Study of COD Removal from the Waste Drilling Fluid and Its Application Chad Oilfield. *J. Water Chem. Technol.* **2021**, *43*, 60-67. https://doi.org/10.3103/S1063455X21010148.
2. Peng, H.; Zou, C.; Wang, C.; Tang, W.; Zhou, J. The effective removal of phenol from aqueous solution via adsorption on CS/β-CD/CTA multicomponent adsorbent and its application for COD degradation of drilling wastewater. *Environ Sci Pollut Res* **2020**, *27*, 33668-33680. https://doi.org/10.1007/s11356-020-09437-1.
3. Zhang, Q.; liu, D.; Liu, Y.; Liu, H.; Huang, M.; Chen, L.; Chen, Q. Screening and characterization of high performance synthetic-based drilling fluids degrading bacteria. *IOP Conf. Ser.: Earth Environ. Sci.* **2020**, *467*, 012143. https://iopscience.iop.org/article/10.1088/1755-1315/467/1/012143/meta.
